# Validation and Application of a Commercial Quantitative Real-Time Reverse Transcriptase-PCR Assay in Investigation of a Large Dengue Virus Outbreak in Southern Taiwan

**DOI:** 10.1371/journal.pntd.0005036

**Published:** 2016-10-12

**Authors:** Huey-Pin Tsai, You-Yuan Tsai, I-Ting Lin, Pin-Hwa Kuo, Kung-Chao Chang, Jung-Chin Chen, Wen-Chien Ko, Jen-Ren Wang

**Affiliations:** 1 Department of Pathology, National Cheng Kung University Hospital, Tainan, Taiwan; 2 Medical Laboratory Science and Biotechnology, National Cheng Kung University, Tainan, Taiwan; 3 Department of Internal Medicine, National Cheng Kung University Hospital, Tainan, Taiwan; 4 Center of Infectious Disease and Signaling Research, National Cheng Kung University, Tainan, Taiwan; 5 National Institute of Infectious Diseases and Vaccinology, National Health Research Institutes, Tainan, Taiwan; Australian National University, AUSTRALIA

## Abstract

**Background:**

Accurate, rapid, and early diagnosis of dengue virus (DENV) infections is essential for optimal clinical care. Here, we evaluated the efficacy of the quantitative real-time PCR (qRT-PCR)-LightMix dengue virus EC kit for DENV detection using samples from a dengue outbreak in Taiwan in 2015.

**Methods:**

Sera from patients with suspected DENV infection were analyzed and compared using the LightMix kit, a Dengue NS1 Ag + Ab Combo kit for detection of NS1 antigen and DENV-specific IgM and IgG antibodies, and an “in-house” qualitative DENV-specific RT-PCR assay.

**Results:**

A total of 8,989, 8,954, and 1581 samples were subjected to NS1 antigen detection, IgM and IgG detection, and LightMix assays, respectively. The LightMix assay yielded a linear curve for viral loads (VL) between 10^2^ and 10^6^ copies/reaction, and the minimum detection limits for DENV serotype 1 (DENV1) and DENV2, DENV3, and DENV4 were 1, 10, and 100 focus forming units (FFU)/mL, respectively. There was 88.9% concordance between the results obtained using the NS1 antigen combo kit and by LightMix analysis, and the diagnostic sensitivity and specificity of the two methods were 89.4 and 100%, and 84.7 and 100%, respectively. Notably, fatal cases were attributed to DENV2 infection, and 79.5% (27/34) of these cases occurred in patients ≥ 71 years of age. Among these older patients, 82.3% (14/17) were NS1/IgM/IgG (+/-/-), exhibiting VLs between 10^6^–10^9^ copies/mL, which was markedly higher than the rate observed in the other age groups.

**Conclusions:**

The LightMix assay was effective for early diagnosis of DENV infection. Our data indicate that high VLs during primary infection in elderly patients may be a positive predictor for severe illness, and may contribute to high mortality rates.

## Author Summary

The LightMix dengue virus EC qRT-PCR assay is effective for early diagnosis of DENV infection. High viral loads during primary infection in elderly patients may comprise a positive predictor for severe illness, and may contribute to high mortality rates.

## Introduction

Dengue virus (DENV) is a member of the viral genus *Flavivirus* (family *Flaviviridae*) that is transmitted by mosquitos, causing endemic and epidemic outbreaks in tropical and subtropical areas. It is estimated that approximately 390 million people are infected with DENV globally each year, resulting in disease manifestation in roughly 96 million patients [1]. DENV infections can either be asymptomatic or manifest with a variety of symptoms, including mild fever, hemorrhagic fever, and fatal shock syndrome [2, 3]. The DENV genome is comprised of a single strand of positive-sense RNA that encodes three structural and seven nonstructural proteins. DENV can be divided into four serotypes (DENV1, 2, 3 and 4), each of which confers partial cross-protective immunity to the other serotypes in humans [4, 5]. Of these, DENV2 is particularly associated with severe dengue illness such as dengue hemorrhage fever; however, high plasma titers of the other serotypes have also been linked to such severe disease [6–9]. Moreover, secondary infection with the other serotypes results in antibody-dependent enhancement (ADE) of disease, leading to more severe dengue illness, and even death [10].

Accurate, rapid, and early diagnosis of DENV infections is important for public health and optimal clinical care. However, the gold standard method of cell culture-dependent isolation of DENV during the acute phase of infection is time-consuming and cumbersome. Detection of the DENV NS1 antigen and DENV-specific nucleic acid sequences during the acute phase is currently widely performed [11]. Additionally, analysis of IgM and IgG antibody responses to dengue virus can reveal the infection period after onset, and can even be applied to discriminate primary and secondary infections. Specifically, an IgM/IgG ratio > 1.7 is indicative of primary infection in certain enzyme-linked immunosorbent assays (ELISA). Meanwhile, patients with secondary infections are characterized as having IgM/IgG ratios < 1.7 or as testing IgG-positive 2 days after onset of illness [12, 13]. Therefore, NS1 antigen, nucleic acid, and antibody (IgM, IgG) screening tests are optimal for dengue diagnosis.

Recently, multiple studies have evaluated real-time reverse transcription (RT)-PCR assays for detection of DENV, the majority of which were developed “in-house” [14–27]. However, the variability in the design of these in-house procedures could yield discordant results for identical samples between facilities. Although commercial available kits provide consistent approaches that could be utilized to overcome this variability, these kits show varying levels of sensitivity and specificity. To ensure the optimal accuracy of laboratory diagnoses, the performance of newly developed methods, including the accuracy, precision, linearity, sensitivity, and specificity of the approach, should be validated.

Between August and November 2015, there were 112 deaths and 22,563 confirmed cases (mortality rate: 0.5%) of DENV2 in a large outbreak in Tainan, Taiwan, as reported by the Taiwan Centers for Disease Control (CDC) [28]. Here, we validate the efficacy of the LightMix dengue virus EC kit, a newly implemented commercial quantitative real-time (qRT)-PCR assay, for the detection of DENV infection in the outbreak.

## Materials and Methods

### Ethics statement

This retrospective study was conducted in Tainan, Taiwan. Approval for the study was obtained from the Institutional Review Board (IRB) of National Cheng Kung University Hospital (No. B-ER-104-228). The demographic and clinical information for the patients were de-linked prior to analysis. Thus, informed consent was not obtained from patients prior to the study.

### Clinical samples

Serum samples from patients with suspected DENV infection were collected at Clinical Virology Laboratory of National Cheng Kung University Hospital (NCKUH) between July and November 2015. Almost all samples were screened via a rapid combo test for antigen and antibody detection for clinical diagnosis; the LightMix dengue virus EC qRT-PCR and serotyping assays were also performed on portions of these samples, per clinical requirements. Patients were categorized as having mild, severe, or fatal cases of dengue according to 2009 World Health Organization (WHO) criteria for severity. For this study, severe dengue cases were considered those that fulfilled one of the following 2009 WHO criteria: severe plasma leakage, severe bleeding, or severe organ involvement.

### NS1 antigen, and IgM and IgG antibody detection

Sera from patients with suspected DENV infection were screened using the one-step immunochromatographic Dengue DuoDengue NS1 Ag + Ab Combo assay (SD BIOLINE, Yongin, Korea). This rapid assay contains two test devices, which can simultaneously detect the levels of NS1 antigen, and IgM and IgG in a sample, respectively, within 15 minutes.

### RNA extraction, reverse transcription, qRT-PCR assay, and serotyping

Viral RNA was extracted from serum samples and an extraction control sample using a QAIamp viral RNA mini kit (Qiagen, Venlo, Netherlands) or the automated extraction system LabTurbo Virus mini Kit in LabTurbo 48 Compact System (Taigen Bioscience Corp., Taipei, Taiwan). qRT-PCR analysis of viral loads was performed using lightMix dengue virus EC kit (qRT-PCR; TIB Molbiol, Berlin, Germany), which is capable of identifying all four dengue serotypes. cDNA was generated from the RNA samples using a FirstStrand cDNA Synthesis kit (Roche, Basel, Switzerland) and a DENV-specific primer included in the LightMix dengue virus EC kit. The qRT-PCR assay was then performed in a LightCycler 2.0 or LightCycler 480 II device (Roche), according to the manufacturer’s instructions; briefly, cDNA was added to a PCR reagent mix containing PCR grade water, the LightMix dengue virus EC kit reagents (including the primers and probes of DENV and the external control), and the mastermix of Roche LightCycler^R^ FastStart DNA Master HybProbe. PCR conditions were as described in the instructions for the LightMix dengue virus EC kit. The amplicon corresponding to the 3'-noncoding region (NCR; 164–187 bp) of DENV was detected using LightCycler Red 640-labeled hybridization probes, while the extraction control (349 bp) derived from Lambda DNA was detected using fluorescence resonance energy transfer (FRET) probes labeled with LightCycler Red 690 (channel 705). The LightMix dengue virus EC kit provides cloned dengue DNA at concentrations of 10^1^ to 10^6^ copies/reaction as standards. The cycle number of the Crossing Point (Cp) of each sample was calculated automatically by the Second Derivative Maximum method (Automated (F" max)), with LightCycler’s software. A logarithmic transformation was applied to all resulting data. The amount of virus per sample (viral load) was reported in copies/reaction, as automatically generated by the LightCycler software.

Serotyping was conducted by an in-house qualitative RT-PCR method, as reported by Tanaka et al. [29].

### Analytical performance of the qRT-PCR assay

The viral reference strains DENV1-8700828 [4 × 10^5^ focus forming units (FFU)/mL], DENV2-454009A (6 × 10^5^ FFU/mL), DENV3-8700829 (4 × 10^5^ FFU/mL), and DENV4-59201818 (2 × 10^5^ FFU/mL) were kindly provided by the Center of Infectious Disease and Signal Research, NCKU.

The accuracy, precision, and analytical sensitivity of the LightMix dengue virus EC kit were evaluated using the reference strains listed above or triplicates of serially diluted plasmid DNA standards (10^1^ to 10^6^ copies/reaction), which were provided with LightMix dengue virus EC kit. Within-run samples were tested in triplicate during the same run, while positive values from another two runs were used for evaluating between-run precision, which represent the coefficient of variation (CV). In parallel, the analytical specificity of the assay was tested using clinical samples containing various viral species. Lastly, the diagnostic sensitivity and specificity of the assays were determined by comparing the results obtained from clinical specimens collected from suspected DENV-infected patients with those obtained by the qRT-PCR assay, in-house qualitative dengue RT-PCR, and NS1 antigen assays.

### Statistical analysis

Frequency distributions for analyses of viral load (VL) levels and the results of NS1 antigen, and IgM and IgG assays were compared by χ^2^ test. *P* values < 0.05 were considered statistically significant. All analyses were conducted using SPSS 17.0 software (SPSS Statistics, Inc., Chicago, IL, USA).

## Results

### Description of the dengue virus outbreak

**Fig 1:**
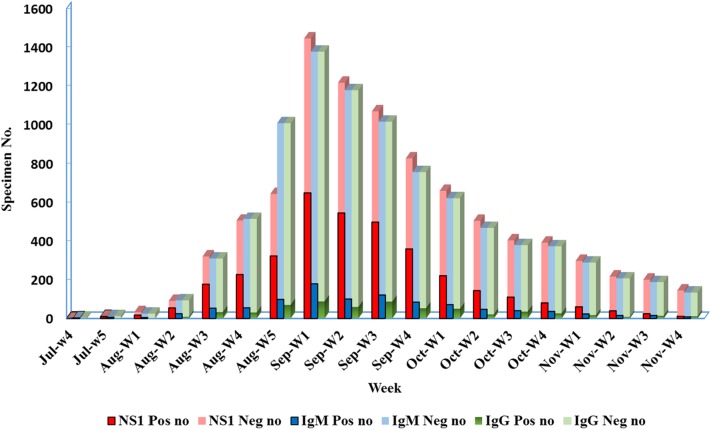
Detection of dengue virus (DENV) NS1 antigen and DENV-specific IgM and IgG antibodies at the Clinical Virology Laboratory of the National Cheng Kung University Hospital (NCKUH) between July and November 2015. (Pos: Positive; Neg: Negative; no: number)

A total of 8,989, 8,954, and 1,581 samples were subjected to NS1 antigen detection, IgM and IgG detection, and qRT-PCR assay analysis, respectively. Of 8,954 samples were screened via a rapid combo test for antigen and antibody detection for clinical diagnosis. Meanwhile, 1,581 specimens collected from patients with suspected DENV infections were simultaneously subjected to NS1 antigen, IgM and IgG, and qRT-PCR assay analyses per clinical requirements. Of these samples, 41.8% (3755/8,989), 11.2% (999/8,954), 6.9% (618/8,954), and 40.2% (619/1,538) tested positive for NS1, IgM, and IgG, and tested positive for DENV in the qRT-PCR assay, respectively. As shown in Fig 1, the peak of the outbreak occurred between the 5^th^ week of August and the 4^th^ week of September (Fig 1).

Of the 44 samples collected from patients with fatal cases (25 female, 19 male) of dengue, 34 tested positive for DENV with the LightMix dengue EC kit, which targets the conserved region of DENV serotypes 1–4. Notably, each of these 34 samples, as well as 67 of the 85 samples obtained from patients diagnosed with severe cases (33 female, 52 male), tested positive for DENV2 by our in-house dengue RT-PCR serotyping assay. Conversely, none of these samples was positive for DENV1, DENV3, or DENV4. The age of the patients associated with fatal disease ranged from 41 years to 91 years, but 42 (95.4%) of the patients were older than 60 years of age.

### Validation of the LightMix Dengue virus EC qRT-PCR assay

**Fig 2:**
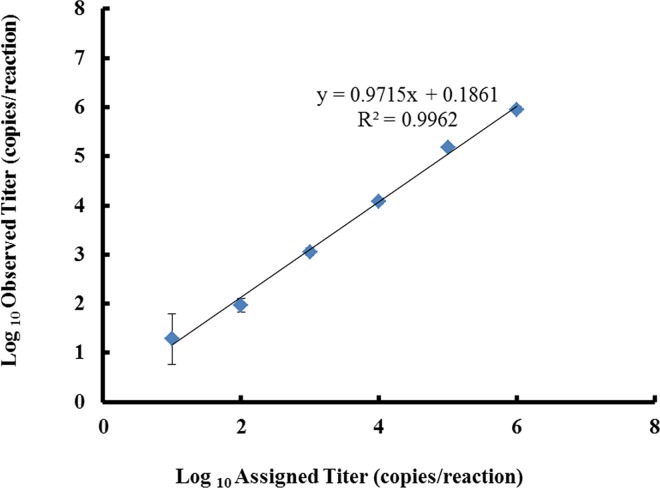
Graphic depiction of the linear range of detection (10^2^ to 10^6^ copies/reaction) of the dengue virus genome by the LightMix Dengue virus EC qRT-PCR assay.

To validate the LightMix Dengue virus EC kit, we evaluated the precision, analytical sensitivity, analytical specificity, clinical sensitivity, and clinical specificity, of assay for diagnosing DENV infection. The between-run and within-run precisions, and the linearity of the assay were evaluated using dilutions of the plasmid DNA standard (10^1^ to 10^6^ copies/reaction) provided with the LightMix kit. While the CVs for the within-run precision when using 10^2^ to 10^6^ plasmid copies/reaction were 0.29%–7.24%, the CV when using 10^1^ copies/reaction was markedly higher (40.29%; Table 1). Meanwhile, the between-run precision values were 1.25%, 1.16%, and 1.22% for 10^2^, 10^4^, and 10^5^ plasmid copies/reaction, respectively (Table 1). Linearity, which indicates the reportable quantifiable range of the assay, was observed between 10^2^ and 10^6^ copies/reaction (R^2^ = 0.99; Fig 2), and the minimum quantification limit (QL) was 10^2^ copies/reaction.

**Table 1:** Within-run and between-run precisions of the LightMix dengue virus EC qRT-PCR assay.

Standard Expected values	Mean	SD	CV
(log_10_ copies/reaction)	(log_10_ copies/reaction)	(log_10_ copies/reaction)	(%)
Within-Run Precision			
6	5.9	0.06	0.98
5	5.2	0.02	0.29
4	4.1	0.06	1.54
3	3.0	0.08	2.57
2	2.0	0.14	7.24
1	1.1	0.52	40.29
Between-Run Precision			
5	5.1	0.06	1.22
4	4.0	0.05	1.16
2	1.9	0.02	1.25

SD, standard deviation; CV, coefficient of variation

For analytical sensitivity testing, quantitative viral reference strains representing the four DENV serotypes were used to spike normal human plasma, adjusted to 10^5^ FFU/mL, and 10-fold serially diluted. The limit of detection (LOD) of the LightMix dengue virus EC qRT-PCR assay for the four subtypes was 1 FFU/mL for DENV1 and DENV2, 10 FFU/mL for DENV3, and 100 FFU/mL for DENV4 (Table 2). Each assay was conducted with 10 replicates, and the positive detection rate was 97.5% (39/40 samples; S1 File).

**Table 2:** Analytical sensitivity test for the LightMix dengue virus EC qRT-PCR assay.

Spiked reference virus	DENV-1	DENV-2	DENV-3	DENV-4
(log_10_ FFU/mL)	(log_10_ copies/mL)	(log_10_ copies/mL)	(log_10_ copies/mL)	(log_10_ copies/mL)
3	6.05	4.89	3.84	4.16
2	4.89	3.74	2.77	2.84
1	3.95	2.51	2.61	NEG
0	2.65	0.63	NEG	NEG
-1	NEG	NEG	NEG	NEG

FFU, focus forming unit

The analytical specificity of the LightMix dengue virus EC qRT-PCR assay was assessed using 20 clinical samples that tested positive by real-time PCR analysis for influenza virus H1N1 or H3N2, cytomegalovirus, human herpesvirus-8 (HHV-8), varicella zoster virus, herpes simplex virus, parvovirus B19, John Cunningham virus (JCV), BK virus, Epstein-Barr virus (EBV), or norovirus. Notably, each of the clinical samples tested negative for DENV, indicating that the presence of these 11 other viruses had no effect on the performance of the LightMix dengue virus EC qRT-PCR assay.

A total of 199 clinical specimens with suspected dengue virus infection were subsequently evaluated simultaneously by both the LightMix dengue virus EC qRT-PCR assay and another in-house dengue-specific RT-PCR assay. The results obtained from each sample were then compared to a composite reference standard, defined as the detection of DENV in one or both assays. The diagnostic sensitivity of the LightMix dengue virus EC qRT-PCR and in-house RT-PCR assay were 99.4% (179/180) and 72.6% (130/180), respectively.

### Comparison of VL levels between various NS1 antigen/IgM/IgG categories

A total of 1,581 specimens collected from patients with suspected DENV infections were simultaneously subjected to NS1 antigen, IgM, IgG, and LightMix dengue virus EC qRT-PCR assay analysis, per clinical requirements, at NCKUH between July 20 and November 30, 2015. Of these specimens, 584 (36.9%) tested positive for DENV in the LightMix dengue virus EC qRT-PCR assay. Meanwhile, among these 585 positive samples, 417 (71.3%) were NS1-positive and IgM/IgG-negative (+/-/-), and contained detectable VLs, of which 274 (274/296; 92.3%) exhibited significantly higher viral copy numbers (10^6^–10^9^ copies/mL; *P* < 0.05) than the specimens in the other NS1/IgM/IgG categories. Furthermore, 71.8% (61/85) and 52.3% (23/44) of the samples in the NS1/IgM/IgG (+/-/-) category collected from patients with severe and fatal dengue contained detectable VLs, respectively, which was higher than the rate observed for other NS1/IgM/IgG categories (*P* < 0.05; Table 3). Notably, of the 855 samples in the NS1/IgM/IgG (-/-/-) category, 7.0% (60) exhibited detectable VLs, which were primarily at <10^6^ copies/mL (51; 85.0%). Furthermore, of the 289 (a + b) samples that were positive for IgM and/or IgG (IgM/IgG: +/-, -/+, +/+), 37.3% (108; c + c2) contained detectable VLs. Meanwhile, VLs of <10^6^ copies/mL were detected in 88.5% (85/96; d/(d + e)) and 75% (9/12; f/ (f + i)) of the NS1-positive and NS1-negative categories, respectively. Of the 104 (j1 + j2) samples that tested positive for NS1 antigen but exhibited no detectable VL, the majority (84; j2; 80.7%) also tested positive for IgM and/or IgG. In contrast, the majority of the samples that tested negative for NS1 antigen but had a detectable VL (60/72; c1/(c1+c2); 83.3%) were negative for IgM and IgG. Of the 996 LightMix dengue virus EC qRT-PCR assay-negative samples, a statistically significant majority (79.8%) were negative for NS1, IgM, and IgG (*P* < 0.05). To investigate the detection efficiency of the NS1 and the LightMix dengue virus EC qRT-PCR assays for DENV during the acute phase of infection, the results obtained from each assay were compared. Notably, the concordance between the two assays was 88.9% (1405/1581; Table 3). Moreover, the diagnostic sensitivity and specificity of the NS1 rapid antigen and LightMix dengue virus EC qRT-PCR assays were 89.4% (625/699) and 100% (882/882), and 84.7% (592/699) and 100%, (882/882), respectively, as assessed using either one or both positive results as the reference standard (S2 File).

**Table 3:** Comparison of viral load levels between various NS1 antigen, IgM, and IgG categories at NCKUH.

Variable	Categories for NS1 antigen/IgM/IgG detection [no. of specimens (%)]								
	+/-/-	+/+/-	+/-/+	+/+/+	-/-/-	-/+/-	-/-/+	-/+/+	No. of total specimens	
**Group**										
Mild dengue case	353 (24.3)	53^a^ (3.7)	50^a^ (3.4)	34^a^ (2.3)	855 (58.9)*	35^b^ (2.4)	33^b^ (2.3)	39^b^ (2.7)	1452 (100)	
Severe dengue case	61 (71.8)*	3^a^ (3.5)	5^a^ (5.9)	15^a^ (17.6)	0 (0.0)	0^b^ (0.0)	0^b^ (0.0)	1^b^ (1.2)	85 (100)	
Fatal dengue case	23 (52.3)*	3^a^ (6.8)	7^a^ (15.9)	10^a^ (22.7)	0 (0.0)	1^b^ (2.3)	0^b^ (0.0)	0^b^ (0.0)	44 (100)	
**Viral load**										
Positive	417^c0^ (71.2)*	39^c^ (6.7)	34^c^ (5.8)	23^c^ (3.9)	60^c1^ (10.3)	2^c2^ (0.3)	4^c2^ (0.7)	6^c2^ (1.0)	585 (100)	
Negative	20^j1^ (2.0)	20^j2^ (2.0)	28^j2^ (2.8)	36^j2^ (3.6)	795^j3^ (79.8)*	34^j3^ (3.4)	29^j3^ (2.9)	34^j3^ (3.4)	996 (100)	
**Positive rate**	95.4%	66.1%	54.8%	39.0%	7.0%	5.6%	12.1%	15%	37.0%	
**Specimen numbers in various viral load levels (copies/mL)**										
<1000	4 (13.8)	6^d^ (20.7)	2^d^ (6.9)	3^d^ (10.3)	10 (34.5)*	0 ^f^ (0.0)	0^f^ (0.0)	4^f^ (13.8)	29 (100)	
10^3^–10^6^	139 (53.7)*	30^d^ (11.6)	26^d^ (10.0)	18^d^ (6.9)	41 (15.8)	1^f^ (0.4)	3^f^ (1.2)	1^f^ (0.4)	259 (100)	
10^6^–10^9^	274 (92.3)*	3^e^ (1.0)	6^e^ (2.0)	2^e^ (0.7)	9 (3.0)	1^i^ (0.3)	1^i^ (0.3)	1^i^ (0.3)	297 (100)	

NCKUH, National Cheng Kung University Hospital; No., number

*P < 0.05 compared to other categories of NS1/IgM/IgG

a: NS1antigen:+, IgM/IgG: one or both positive

b: NS1 antigen:-, IgM/IgG: one or both positive

c0: NS1 antigen/IgM/IgG/VL:+/-/-/+

c: NS1 antigen/VL:+/+, IgM/IgG: one or both positive

c1: NS1 antigen/IgM/IgG/VL:-/-/-/+

c2: NS1 antigen/VL:-/+, IgM/IgG: one or both positive

j1: NS1 antigen/IgM/IgG/VL:+/-/-/-

j2: NS1 antigen/VL:+/-, IgM/IgG: one or both positive

j3: NS1 antigen/VL:-/-

d: NS1 antigen/VL (<10^6^):+/+, IgM/IgG: one or both positive

f: NS1 antigen/VL (<10^6^):-/+, IgM/IgG: one or both positive

e: NS1 antigen/VL (10^6^−10^9^):+/+, IgM/IgG: one or both positive

i: NS1 antigen/VL (10^6^−10^9^):-/+, IgM/IgG: one or both positive

Among the 34 (a1 + a + b) LightMix dengue virus EC qRT-PCR assay-positive specimens collected from the 44 patients with fatal dengue, half (17; a1 + a) had VLs of 10^6^–10^9^ copies/mL (Table 4). Meanwhile, 35 (c1 + c) of the 53 (c + c1 + d), 66% LightMix dengue virus EC qRT-PCR assay-positive NS1/IgM/IgG (+/-/-) specimens collected from patients with severe dengue exhibited VLs of 10^6^–10^9^ copies/mL. Notably, such high VLs were also detected in 82.3% (14/17; a1/(a1 + a)) and 57.1% (20/35; c1/(c1 + c)) of the NS1/IgM/IgG (+/-/-) specimens collected from patients with fatal and severe dengue aged ≥71 years, respectively. Although 10 samples from fatal cases and 17 samples from severe cases were qRT-PCR-negative for DENV, these patients were still diagnosed with dengue infection, as they were positive for either NS1 antigen or IgM/IgG (Table 4).

**Table 4:** Comparison of age groups between various NS1 antigen/IgM/IgG and viral load categories among fatal and severe cases.

NS1/IgM/IgG	Fatal cases (n = 44)							Severe cases (n = 85)						
	+/-/-			+/(+/-, -/+, +/+)			-/+/-	+/-/-			+/(+/-, -/+, +/+)			-/(+/+)
viral load levels*	10^6^−10^9^	10^3^−10^6^	Neg**	10^6^−10^9^	10^3^−10^6^	Neg	Neg	10^6^−10^9^	10^3^−10^6^	Neg	10^6^−10^9^	10^3^−10^6^	Neg	Neg
<40 years old	0	0	0	0	0	0	0	5^c^	0	2	0	0	0	0
41–50 years old	1^a^	0	0	0	0	0	0	2^c^	2^d^	0	0	1	0	0
51–60 years old	0	0	0	0	1^b^	0	0	4^c^	1^d^	0	1	2	0	0
61–70 years old	2^a^	2^b^	0	0	1^b^	2	0	4^c^	4^d^	0	0	0	1	0
≥71 years old	14^a1^	3^b^	1	0	10 ^b^	6	1	20^c1^	11^d^	5	1	10	8	1

*copies/mL

**Negative

a: 41–70 years old of the patients with fatal dengue had VLs of 10^6^–10^9^ copies/mL

a1: ≥71 years old of the patients with fatal dengue had VLs of 10^6^–10^9^ copies/mL

b: fatal cases had VLs of 10^3^–10^6^ copies/mL

c: <70 years old of the patients with severe dengue had VLs of 10^6^–10^9^ copies/mL

c1: ≥71 years old of the patients with severe dengue had VLs of 10^6^–10^9^ copies/mL

d: severe cases with NS1 antigen/IgM/IgG (+/-/-) had VLs of 10^3^–10^6^ copies/mL

## Discussion

Currently, detection of DENV-specific nucleic acids during the acute phase of infection is widely applied for dengue diagnosis. Indeed, many studies have evaluated the efficacy of real-time RT-PCR assays for the detection of DENV. The results of these previously published reports are summarized in Table 5. The majority of the studies focused on in-house and TaqMan assays, as opposed to commercial SYBR and FRET assays. Additionally, the most popular target region used for detection was the 3'-NCR. Meanwhile, the 3'-NCR and NS5 regions, as well as combinations of different genes, have commonly been utilized as target regions for serotyping [14–16, 19–23, 25–27, 30–32]. Although Levi et al. (2007) observed high levels of agreement between the commercial RealArt real-time RT-PCR kit and an in-house multiplex RT-PCR for detection of DENV3 (and occasionally DENV2), the sensitivity of these published methods are highly diverse (3.8–100%) [33]. Moreover, among the 37 laboratories that participated in an international external quality control assessment study to evaluate various dengue-specific RT-PCR assays, 80.4% reported a lack of sensitivity for dengue molecular diagnosis [34]. In this study, we detected DENV in 100% (102/102) of the DENV2-positive samples by the LightMix dengue virus EC qRT-PCR assay, compared to only 66.7% (68/102) of the same samples by another in-house qualitative serotyping RT-PCR assay. To date, this is the first report to evaluate a commercially available kit for detection and quantification of DENV via FRET and real-time PCR analyses.

**Table 5:** Comparison of published real-time reverse transcriptase-polymerase chain reaction methods for detection of dengue virus.

Target region	Quantitation	TaqMan probe	Sensitivity (%)	References
**1.** **In-house dengue grouping assay**				
3'-NCR	No	Yes	79.4 (121/153)	[11]
	Yes	Yes	3.8 (3/79)	[25]
	No	Yes	98.5 (66/67)	[14]
	Yes	No (SYBR)	88 (46/52)	[24]
NS1	Yes	Yes	100 (26/26)	[35]
5'-NCR+capsid	Yes	Yes	98 (196/200)	[32]
**2.** **In-house dengue serotyping assay**				
3'-NCR	Yes	No (FRET)	Unknown	[24]
	No	No (SYBR)	91 (30/33)	[16]
	Yes	Yes	92.8 (115/125)	[21]
	Yes	Yes	94.4 (17/18)	[30]
	Yes	Yes	100 (39/39)	[20]
C-prM	No	No (SYBR)	100 (33/33)	[16]
DENV1-NS5, DENV2-E, DENV3, 4-prM,	No	Yes	97.92(83/86)	[36]
DENV1-NS5, DENV2-E protein, DENV3-NS1, DENV4-5'-NCR/capsid	Yes	Yes	100 (85/85)	[19]
NS5	Yes	Yes	97.2 (35/36)	[26]
	No	Yes	91 (70/77)	[16]
	Yes	Yes	89.5 (337/376)	[23]
Envelope gene	No	Yes	100 (35/35)	[15]
Capsid	Yes	Yes	Unknown	[37]
5'-NCR+capsid	Yes	Yes	Unknown	[25]
prM and E region	No	Yes	Unknown	[22]
NS5 and C region	No	Yes	92.5 (62/67)	[14]
**3.** **Commercial kit: target gene non-specific**				
InnuDETECT Dengue TwoStep assay	Unknown	Unknown	44.4 (52/117)	[38]
AbTES DEN 5 qPCR	Yes	Unknown	97.4 (114/117)	[38]
Geno-Sen’s dengue 1–4 real-time RT-PCR kit	Yes	Yes	85.2 (117/138)	[39]
Liferiver Dengue virus general type realtime RT-PCR	Yes	Yes	Unknown	[39]
Realstar dengue RT-PCR kit 1.0 (Altona, Germany)	No	Yes	83.3 (112/135)	[39]
			72.3 (110/151)	[31]
RealArt Dengue 1–4 realtime PCR kit (QAIGEN, Germany)	Yes	Yes	37 (75/203)	[33]
**4.** **Commercial Simplexa dengue RT-PCR kit (Focus, CA)-**target gene specify on DENV1-NS5S, DENV2-NS3, DENV3-NS5, DENV4-capsid	No	Yes	93.2 (140/151)	[39]
**5.** **Commercial LightMix dengue virus EC viral load kit** 3'-NCR	Yes	No (FRET)	84.7 (592/699) (compared to NS1 Ag)	This study
			99.4 (179/180) (compared to in-house RT-PCR)	This study

NCR, non-coding region; FRET, fluorescence resonance energy transfer; DENV, dengue virus

A linear curve for the LightMix dengue virus EC qRT-PCR assay was obtained between 10^2^ and 10^6^ copies/reaction (R^2^ = 0.99). Notably, the assay was more sensitive for detection of DENV1 and DENV2 than for DENV3 and DENV4. Such differences in sensitivity for distinct viral serotypes have been reported in the other studies [22, 24]. Indeed, these differences were expected because of the free noninfectious virus particles present in the virus stocks, which were quantitated by plaque assay analysis, and because each assay was limited by its primer and probe design.

Therefore, we evaluated the diagnostic sensitivity of the assay to validate its use for detection of DENV in specimens derived from infected patients. For these analyses, we compared the results obtained by NS1 antigen, IgM, IgG, and LightMix dengue virus EC qRT-PCR assay analyses of samples collected from suspected DENV-infected patients during an outbreak in Taiwan in 2015. The NS1/IgM/IgG (+/-/-) category was associated with the highest proportion (274/417; 65.7%) of samples exhibiting high VLs (10^6^–10^9^ copies/mL). Meanwhile, the highest percentage (88.5%) of specimens with medium VLs (<10^6^ copies/mL) was observed in the NS1-positive and IgM and/or IgG-positive category. Notably, the highest ratios (80.7%; 84/104; j2/(j1 + j2)) of antibody-positive and -negative specimens (83.3%, 60/72; c1/(c1 + c2)) were observed in the NS1/VL (+/-) and NS1/VL (-/+) categories, respectively (Table 3). These data indicate that NS1 antigen can remain within the human sera even after antibody-mediated reduction or clearance of the virus. Consistent with this conclusion, Hernandez et al. (2013) [40] previously demonstrated that while dengue IgM antibody-negative patients exhibit significantly higher VLs than IgM-positive patients, the presence of IgM does not correlate with NS1 antigen clearance. Recently, Tittarelli et al. (2016)[13] found no significant differences in VL between patients with primary and secondary infections. In this study, 26 of the 34 specimens in the NS1/IgM/IgG/VL (+/-/+/+) category (76.4%) had VLs of 10^3^–10^6^ copies/mL, indicating the presence of a suspected secondary infection group with medium VLs among the patients examined during this outbreak. In contrast, Laue et al. (1999) reported >5 × 10^6^ copies of DENV RNA in samples collected from two suspected dengue secondary infection patients that were IgG-positive but IgM-negative on the first and second day after the onset of illness [30]. The results of our study suggest that secondary infections associated with medium VL may be modulated by IgG antibody. The correlations between severe dengue disease and high VL, secondary infection, and DENV2 infection have been discussed in multiple studies [8, 9, 41]. According to data collected by the Taiwan CDC (Centers for Disease Control), 95% of the fatal cases in this outbreak presented with comorbidities such as hypertension (72.9%), diabetes (47.7%), chronic kidney disease (31.8%), heart diseases (17.8%), and cancer (11.2%) [28]. Therefore, we found that high VLs of DENV2 contributed to the high mortality rates observed in elderly patients in primary DENV infections. Patients with comorbidities may also play a role which needs to be examined further.

Ahmed et al. (2014) observed maximal sensitivity of the NS1 antigen and real time RT-PCR assays for DENV detection on the second and third day of illness, respectively [11]. The group therefore recommended the application of these two rapid and efficient assays as early diagnostic tests during the acute phase of infection. In this study, there was concordance between the results obtained using these two methods. Furthermore, the diagnostic sensitivity of the two assays was above 80%. Additionally, while a high proportion of the 617 (c0+c+j1+j2) NS1-positive specimens was also VL-positive (513/617, (c0+c)/(c0+c+j1+j2), 83.1%), only 72 of the 964 (72/964, (c1+c2)/(c1+c2+j3), 7.4%) NS1-negative specimens were VL-positive. These data indicate that NS1 antigen detection could be used for rapid diagnostic screening during large outbreaks, while the LightMix dengue virus EC qRT-PCR assay could be employed as a supplemental test for analysis of specimens that test NS1-negative during the acute phase of infection.

There were limitations to our study. Notably, the NS1/IgM/IgG (+/-/-) category contained 20 VL-negative specimens (Table 3), which might indicate the presence of false-negative results owing to variant virus or virus-free in sera. As such, the use of primers or probes to detect additional DENV target regions, and the evaluation of peripheral blood mononuclear cells instead of sera could be considered alternative methods for confirming DENV diagnoses. Accurate, rapid, and early diagnosis of DENV infections could reduce the risk of development of life-threatening dengue illness, particularly in NS1/IgM/IgG-negative cases. Our results indicate that this LightMix dengue virus EC qRT-PCR assay could be helpful in predicting mortality in primary infected elderly patients, for early diagnosis of DENV infections, and for controlling future dengue outbreaks.

## Supporting Information

S1 Checklist STARD checklist and STARD flowchart. (DOCX)

S1 File Positive rate among DENV 1–4 LOD replicates (LOD: limit of detection). (XLSX)

S2 File Diagnostic sensitivity and specificity of the NS1 rapid antigen and LightMix dengue virus EC qRT-PCR assays. (XLSX)
